# Neonates With Complex Cardiac Malformation and Congenital Diaphragmatic Hernia Born to SARS-CoV-2 Positive Women—A Single Center Experience

**DOI:** 10.1177/2150135120950256

**Published:** 2020-08-27

**Authors:** Nimrod Goldshtrom, Diana Vargas, Angelica Vasquez, Faith Kim, Kinjal Desai, Mariel E. Turner, Oliver Barry, Alejandro Torres, Stéphanie Levasseur, Svetlana Strletsova, Palka R. Gupta, Jennifer R. Defazio, Vincent Duron, William Middlesworth, Lisa Saiman, Russell Miller, Dena Goffman, Emile A. Bacha, David Kalfa, Damien J. LaPar, Ganga Krishnamurthy

**Affiliations:** 1 Department of Pediatrics, New York-Presbyterian/Morgan Stanley Children’s Hospital, 21611 Columbia University Irving Medical Center, New York, NY, USA; 2 Department of Nursing, New York-Presbyterian/Morgan Stanley Children’s Hospital, 21611 Columbia University Irving Medical Center, New York, NY, USA; 3 Department of Surgery, 21611 New York-Presbyterian/Morgan Stanley Children’s Hospital, Columbia University Irving Medical Center, New York, NY, USA; 4 Department of Infection Prevention and Control, 21611 New York-Presbyterian/Morgan Stanley Children’s Hospital, Columbia University Irving Medical Center, New York, NY, USA; 5 Department of Obstetrics and Gynecology, 21611 New York-Presbyterian/Morgan Stanley Children’s Hospital, Columbia University Irving Medical Center, New York, NY, USA

**Keywords:** COVID-19, SARS-CoV-2, congenital heart disease, neonate, vertical transmission

## Abstract

**Background:**

Our understanding of the impact of severe acute respiratory syndrome coronavirus 2 (SARS-CoV-2) on pregnancies and perinatal outcomes is limited. The clinical course of neonates born to women who acquired coronavirus disease 2019 (COVID-19) during their pregnancy has been previously described. However, the course of neonates born with complex congenital malformations during the COVID-19 pandemic is not known.

**Methods:**

We report a case series of seven neonates with congenital heart and lung malformations born to women who tested positive for SARS-CoV-2 during their pregnancy at a single academic medical center in New York City.

**Results:**

Six infants had congenital heart disease and one was diagnosed with congenital diaphragmatic hernia. In all seven infants, the clinical course was as expected for the congenital lesion. None of the seven exhibited symptoms generally associated with COVID-19. None of the infants in our case series tested positive by nasopharyngeal test for SARS-CoV-2 at 24 hours of life and at multiple points during their hospital course.

**Conclusions:**

In this case series, maternal infection with SARS-CoV-2 during pregnancy did not result in adverse outcomes in neonates with complex heart or lung malformations. Neither vertical nor horizontal transmission of SARS-CoV-2 was noted.

## Introduction

In less than six months, the coronavirus disease 2019 (COVID-19) pandemic has swiftly spread from one city in China to over 190 countries across six continents.^1,2^ As of this writing, the number of confirmed infections has surpassed 4.5 million and fatalities are now in excess of 300,000.^1,2^ New York City (NYC) emerged as the epicenter of the pandemic in the United States.^2^ Infections surged exponentially in NYC in late March and early April 2020, with more than 10,000 new cases per day at the peak of the pandemic.^2^ Within three weeks of the first identified COVID-19 patient on March 1, thousands of infected individuals sought medical care in NYC, including pregnant women.

Pregnant women infected with the Severe Acute Respiratory Syndrome Coronavirus 1 (SARS-Cov-1) and Middle East Respiratory Syndrome (MERS) coronaviruses were at greater risk of severe infection and adverse perinatal outcomes compared to women who were not pregnant at the time of infection.^3–5^ Our knowledge about the impact of SARS-CoV-2 infection, the etiological agent causing COVID-19, on pregnant women and their newborn infants is limited. Neonatal infections with SARS-CoV-2 have been described although robust data on vertical transmission are lacking. In most instances where neonatal infection has been reported, close contact with infected mother or caregiver is postulated to have occurred.^6–21^ Miscarriage, intrauterine fetal demise, and premature birth have been reported with COVID-19 while most neonates who acquire SARS-CoV-2 infections have either been asymptomatic or experienced a mild course.^6–21^ There are currently no reports on the impact of COVID-19 on pregnancies complicated by complex malformations in the fetus nor the clinical and hospital course of these neonates after birth. In this case series, we report on our experience at a single large medical center in NYC, with newborn infants diagnosed with complex congenital malformations born to mothers who acquired COVID-19 during pregnancy.

## Methods

### Setting

This study was conducted at New York Presbyterian’s Morgan Stanley Children’s Hospital and Columbia University Irving Medical Center, a quaternary care hospital that predominantly serves northern Manhattan and neighboring boroughs including Westchester county and New Jersey. Patients were admitted to the Infant Cardiac Unit, a 17-bed intensive care unit dedicated to the care of neonates and young infants with congenital heart disease. The Institutional Review Board of Columbia University has approved this study under expedited review with waiver of informed consent.

### Data Source

Eligible patients were identified from the Infant Cardiac Unit’s administrative database. Data were obtained by manual chart review and included neonates born to mothers who were diagnosed with COVID-19 between March 16, 2020, and May 7, 2020. Data collected included maternal and infant demographic information, maternal and infant SARS-CoV-2 test results, maternal symptoms, timing of maternal clinical presentation, mode of delivery, maternal disposition, Apgar scores, resuscitation at birth, cardiac and other diagnoses, hospital course, and disposition of neonates.

### Study Population

This case series included neonates with prenatal diagnosis of complex congenital malformations of the heart or lung born to women who tested positive by nasopharyngeal swab for SARS-CoV-2 between March 16, 2020, and May 7, 2020. Infants born to women with symptoms suggestive of COVID-19, but not confirmed by laboratory test, were not included.

A dedicated multidisciplinary COVID-19 team from the Infection Prevention and Control (IP&C) department provided guidance on infection prevention and control measures, breast milk and breastfeeding practice, as well as guidance on timing and frequency of testing and visitation to our unit and hospital.

SARS-CoV-2 testing: All neonates underwent testing for SARS-CoV-2 by nasopharyngeal swab at 24 hours, at 14 days, and in-between or after if symptoms emerged or if there was a scheduled procedure when a repeat test was performed 24 hours prior to the procedure as per institutional guidelines. Cobas (Roche) or Xpert Xpress (Cepheid) Reverse transcription polymerase chain reaction (RT-PCR) assay were used to test for neonates for SARS-CoV-2.

### Isolation Precautions

Based on Centers for Disease Control and Prevention and local IP&C interim guidance, neonates born to COVID-19 positive mothers were considered persons under investigation (PUI). While a PUI, newborns were placed on droplet and contact isolation in negative pressure rooms until infection in the neonate was excluded at the end of the incubation period, that is, 14 days. Airborne precautions were implemented if aerosol-generating procedures such as continuous positive airway pressure (CPAP), oral suctioning, or intubation were performed.

For neonates born at our hospital, attendance at delivery was organized to minimize exposure of staff and preserve personal protective equipment (PPE) while maintaining adequate numbers of personnel to provide effective resuscitation. Hospital policies were followed to ensure staff protection with appropriate PPE usage. After initial stabilization in the delivery room, transport of these neonates was accomplished by placing the infants in an enclosed incubator with an additional team outside of the delivery room or obstetrical operating room. The transport team wore appropriate PPE, was accompanied by support staff to facilitate movement through hospital, and transported patients immediately to a negative pressure room in the Infant Cardiac Unit at our hospital.

### Visitation Policy

During the pandemic, visitation has been restricted to the Infant Cardiac Unit. For all infants, only one designated, asymptomatic parent or guardian was permitted, all of whom wore a gown, gloves, and a surgical face mask. Mothers who tested positive for SARS-CoV-2 were prohibited from entering the Infant Cardiac Unit until the local IP&C team cleared her for visitation. Clearance parameters generally included an afebrile period of three consecutive days without antipyretics and at least 14 days (seven days if asymptomatic) had passed since the onset of symptoms and other symptoms had markedly improved.

### Breast Milk and Feeding

Symptomatic mothers who were not yet cleared for visitation were encouraged to express breast milk at home, store appropriately, and deliver to the Infant Cardiac Unit. Per hospital policies and CDC recommendations, safe expression of breast milk in symptomatic mothers could be performed while mothers wore a mask, performed good breast and hand hygiene, and cleaned the pump between each session.^22^ Asymptomatic mothers who were cleared for visitation were allowed to breastfeed or provide pumped milk to their child while wearing a mask and having performed good hand and breast hygiene.

### Statistical Procedures

Descriptive statistics and data collection were performed using Microsoft Excel 2016 MSO (16.0.4266.1001). Parametric results were expressed as mean and standard deviation (SD). Nonparametric results were expressed as median and interquartile range (IQR).

## Results

Between March 16 and May 7, 2020, seven neonates with complex congenital heart and lung malformations born to women who tested positive for SARS-CoV-2 were admitted to the Infant Cardiac Unit at our institution. Notably, of the seven, three women were asymptomatic during their hospital course and denied prior history of COVID-19 symptoms. Three women experienced mild to moderate symptoms of COVID-19 prior to delivery and tested positive between five and eleven days prior to delivery. One woman who was asymptomatic at admission to the labor and delivery ward developed symptoms of COVID-19 immediately after delivery and tested positive for SARS-CoV-2. During this period, approximately 15% of women who delivered at our hospital tested positive for SARS-COV-2.

Table 1 shows demographic and clinical results. Five neonates were born at our hospital and two were transferred from other institutions. Diagnosis of cardiac or lung malformation was established prenatally in all neonates. Four male and three female infants were born between 34 4/7 and 40 weeks’ gestation (median: 38 5/7weeks, IQR 37-39 weeks). Mean and SD for birthweight were 2,845 ± 485 g. One-minute Apgar scores ranged from 3 to 9, with 5-minute Apgar scores of 7 to 9. Five neonates received expressed breast milk as either part or all of their enteral nutrition during admission.

**Table 1.** Clinical Characteristics of Infants.

	Cases						
	1	2	3	4	5	6	7
Trimester of maternal presentation	3	3	3	3	3	3	3
Maternal status	+	+	–	+	–	–	+
Maternal PCR swab result	+	+	+	+	+	+	+
Gestational age at delivery (week days)	39 0/7	36 4/7	38 5/7	40 0/7	38 2/7	39 0/7	34 4/7
Gender	Male	Male	Female	Male	Female	Male	Female
Birthweight (grams)	3320	2485	2580	3195	2400	3530	2405
Apgar score (1-min, 5-min)	8, 8	3, 7	8, 9	9, 9	8, 8	8, 8	9, 9
Mode of delivery	NSVD	C/S	NSVD	C/S	NSVD	NSVD	C/S
Clinical course	d-TGA/IVS, s/p BAS and repair, discharged home	L-CDH, VA-ECMO (3 days), s/p repair, recovering	d-TGA/PA/VSD, Dandy-Walker, s/p PDA stent	PA/IVS, s/p balloon dilatation, PDA stent, discharged home	Critical PS s/p balloon dilatation, discharged home	Critical PS s/p balloon dilatation, recovering	l-TGA, PS, 2:1 heart block. Feeding and growing.
Enteral nutrition	EBM	EBM, PHDM, Formula	Formula	EBM, PHDM, Formula	EBM, Formula	EBM	PHDM
Infant SARS-CoV-2 PCR (nasal swab)	–	–	–	–	–	–	–

Abbreviations: BAS, balloon atrial septostomy; C/S, cesarean section; d-TGA, dextro-transposition of the great arteries; EBM, expressed breast milk; IVS, intact ventricular septum; L-CDH, left congenital diaphragmatic hernia; l-TGA, levo-transposition of the great arteries; NA, not applicable; NSVD, normal spontaneous vaginal delivery; PA, pulmonary atresia; PCR, polymerase chain reaction; PDA, patent ductus arteriosus; PHDM, pasteurized human donor milk; PS, pulmonary stenosis; VA-ECMO, venous arterial extracorporeal membrane oxygenation; VSD, ventricular septal defect.

### Cases

#### Case 1

This is a full-term male neonate born at our institution with a prenatal diagnosis of d-transposition of the great arteries with intact ventricular septum and a restrictive atrial communication. The patient’s mother developed respiratory symptoms and fever two-and-a-half weeks prior to delivery and was diagnosed with COVID-19 11 days prior to delivery. After birth and immediate stabilization, balloon atrial septostomy was performed in a negative pressure room in the Infant Cardiac Unit. Arterial switch operation was performed on day of life (DOL) 4. His postoperative course was unremarkable, and he was discharged home on DOL 13. SARS-CoV-2 tests on this infant on DOL 1, 2, 4, and 8 were negative.

#### Case 2

This is a late preterm male neonate born at our institution with prenatal diagnosis of left congenital diaphragmatic hernia (CDH). Prenatal magnetic resonance imaging confirmed left-sided defect containing stomach, bowel and liver, a lung area-to-head circumference ratio (LHR) of 0.7, and observed-to-expected LHR ratio of 0.25. Based on these reports, the infant was at higher risk for needing extracorporeal membrane oxygenation (ECMO) in the postnatal period. Although his mother developed mild symptoms and tested positive one week prior to delivery, she presented on the day of admission with worsening respiratory distress and a nonreassuring fetal heart tracing prompting delivery. On DOL 2, the neonate required escalation of support for hypoxic respiratory failure and pulmonary hypertension and was placed on veno-arterial ECMO for three days. The ECMO course was complicated by coagulopathy prompting decannulation on DOL 4. Gortex patch repair of the CDH defect was accomplished on DOL 5 without complications. SARS-CoV-2 tests on DOL 1, 4, and 14 were negative. At the time of this writing, this neonate is recovering well, weaning off noninvasive respiratory support, and establishing feeding skills.

#### Case 3

This is a full-term female neonate born at our institution with complex congenital anomalies including d-transposition of the great arteries, pulmonary atresia, ventricular septal defect, Dandy Walker malformation, micro/retrognathia, glossoptosis, and intrauterine growth restriction. A postnatal diagnosis of trisomy 13 was established. A stent in the ductus arteriosus was placed on DOL 10. This patient is currently in the Infant Cardiac Unit on nasal CPAP, awaiting tracheostomy for upper airway obstruction. SARS-CoV-2 tests on DOL 1, 7, 9, and 14 were negative.

#### Case 4

This is a term male neonate born at an outside institution with pulmonary atresia and intact ventricular septum who underwent radiofrequency perforation and balloon dilatation of the pulmonary valve on DOL 10. Due to the inability to wean off from Prostaglandin E1 (PGE-1) infusion, this patient underwent ductal stent placement on DOL 19. SARS-CoV-2 tests on DOL 1, 5, 9, 15, and 18 were negative. This neonate was discharged home on DOL 29.

#### Case 5

This is a term female neonate born at our institution with prenatal diagnosis of critical pulmonary stenosis who underwent balloon dilatation of the pulmonary valve on DOL 2. SARS-CoV-2 testing on DOL 1 was negative. This neonate was discharged home on DOL 7 without additional SARS-CoV-2 testing.

#### Case 6

This is a term male neonate born at our institution with prenatal diagnosis of pulmonary atresia with intact ventricular septum and postnatal diagnosis of critical pulmonary stenosis. This patient underwent balloon dilatation of the pulmonary valve on DOL 4. Currently, this patient remains in the intensive care unit and is weaning from CPAP and PGE-1. SARS-CoV-2 tests performed on DOL 1 and 3 were negative.

#### Case 7

This is a preterm female infant born at an outside institution at 34 4/7 weeks’ gestation due to placenta previa, vaginal bleeding, and nonreassuring fetal heart tracings. Cardiac diagnoses included l-transposed great arteries, large ventricular septal defect, mild pulmonary stenosis, and 2:1 atrioventricular block. This infant was placed on CPAP and weaned to room air at term gestation. No cardiac intervention was performed. She was transferred back to the referring institution to improve feeding skills prior to discharge. All SARS-CoV-2 tests were negative on DOL 2, 5, 14, and 23.

## Discussion

The clinical course of neonates with complex cardiac or lung malformations born to women with COVID-19 has not been described previously. Adverse pregnancy outcomes including preterm births have been reported with COVID-19.^6–21^ In this series, except for two, all neonates were born at term gestation. In case 2, the infant was born early primarily due to symptoms in the mother leading to fetal intolerance of labor. In case 7, early delivery may have been related to maternal COVID-19 diagnosis. All seven neonates in our case series followed a hospital course that appears comparable to patients with similar diagnoses cared for at our institution in the pre-pandemic era.

In all seven neonates, infection in the mother occurred in the late third trimester. In six cases, delivery occurred 5 to 11 days after testing positive for COVID-19 with one case of postnatal maternal diagnosis. We are only now beginning to understand the impact of SARS-CoV-2 infection on pregnancy outcomes if the infection occurred earlier in pregnancy, including in the first or second trimesters. Rates of spontaneous abortion and fetal growth restriction in early trimester maternal infection do not appear increased in a recent report.^23^ In the same study, while the risk of preterm birth (<37 weeks’ gestation) was increased, spontaneous preterm birth was not. We do not yet know the impact of severe COVID-19 on the fetus if delivery occurs remote from infection. During the 2009 H1N1 influenza pandemic, 25% of neonates who were delivered after the mother had a hospital discharge from severe or critical illness were born small for gestational age and of lower birth weight than the general population.^24^ During the SARS epidemic, neonates delivered several weeks after maternal infection experienced intrauterine growth restriction.^3^

In general, vertical transmission can occur from mother to newborn antepartum across the placenta, intrapartum during the birthing process, or after birth through the ingestion of breast milk.^25^ In our series, nasopharyngeal swab test for SARS-CoV-2 was negative at 24 hours and at multiple points thereafter including on DOL 14 when neonatal infection was finally excluded in all seven cases. The usual routes of transmission of SARS-CoV-2 are by respiratory droplets, by close contact with infected individuals, or through fomite transmission from contaminated surfaces or objects.^26^ The mode(s) of transmission of SARS-CoV-2 to the newborn has not been clearly defined, and the potential contributions of antepartum and intrapartum infection are unclear. Although placental invasion of SARS-CoV-2 has been reported, robust data that support in utero transmission are lacking.^27–29^ Studies have detected immunoglobulin M in newborns suggestive of intrauterine infection, although the sensitivity and specificity of these tests are unknown.^17^ Newborns can also potentially acquire infection through horizontal transmission from infected contacts.^11–13^ Thus far, to our knowledge, there are published reports of 28 neonates who have tested positive for SARS-CoV-2.^6–21^ While the modes of transmission to the neonate have not been clearly outlined in these reports, close contact with the infected mother or other caregiver is presumed.^11–13^ In our case series, all of the seven infants were separated from the mother for the entire duration of the incubation period, that is, 14 days and have tested negative on multiple tests. This is a specific practice we employed in our Neonatal intensive care unit and Infant Cardiac Unit and not our standard well baby nursery. Our hospital IP&C guidelines restricting maternal visitation to Neonatal Intensive Care Unit and Infant Cardiac Unit infants has likely prevented horizontal transmission to these infants.

In our case series, four of the seven women did not exhibit any symptoms of COVID-19 at the time of arrival to the labor and delivery ward, and three continued to be asymptomatic during the entire hospital course. Based upon the high asymptomatic carriage rate and the highly efficient manner in which this virus is transmitted, we think it is prudent to test all pregnant women who present for delivery for SARS-CoV-2 infection based on local prevalence and resources.^7^

This study has several limitations. It is a small case series of seven patients from an academic University hospital at the epicenter of the global pandemic in the United States. Although our results are in accordance with the majority of reports of vertical transmission, large data sets and data registries are needed to confirm the low probability of vertical transmission of SARS-CoV-2. Additionally, this study does not provide data on pregnant women with fetuses with complex cardiac or lung malformations who were positive for SARS-CoV-2 and experienced either miscarriage or intrauterine fetal death.

In conclusion, neonates with complex cardiac or lung malformation born to women diagnosed with COVID-19 during pregnancy followed an unremarkable course. We did not identify vertical transmission in our series. Additionally, we did not experience any horizontal transmission from mothers with COVID-19.

## Abbreviations and Acronyms

CDH: congenital diaphragmatic hernia
COVID-19: coronavirus disease 2019
CPAP: continuous positive airway pressure
DOL: day of life
ECMO: extracorporeal membrane oxygenation
IP&C: Infection Prevention and Control
IQR: interquartile range
LHR: lung area-to-head circumference ratio
NYC: New York City
PGE-1: Prostaglandin E1
PPE: personal protective equipment
PUI: persons under investigation
SD: standard deviation
